# Use of percutaneous microwave ablation for the treatment of bone tumors: a retrospective study of clinical outcomes in 47 patients

**DOI:** 10.1186/s40644-019-0275-8

**Published:** 2019-12-18

**Authors:** Min-hao Wu, Ling-fei Xiao, Fei-fei Yan, Shi-Liang Chen, Chong Zhang, Jun Lei, Zhou-ming Deng

**Affiliations:** Department of Spine Surgery and Musculoskeletal Tumor, Zhongnan Hospital of Wuhan University, 168 Donghu Street, Wuchang District, Wuhan, 430071 Hubei People’s Republic of China

**Keywords:** Bone tumor, Microwave ablation, Surgery, Minimally invasive, Clinical efficacy

## Abstract

**Objective:**

The present study aimed to evaluate the short-term clinical performance and safety of percutaneous microwave ablation (MWA) techniques for the treatment of bone tumors.

**Methods:**

This single-institution retrospective study investigated 47 cases of bone tumors treated by MWA from June 2015 to June 2018. The study included 26 patients (55.3%) with benign bone tumors and 21 patients (44.7%) with malignant bone tumors. The tumors were located in the spine or sacrum (15, 31.9%), the upper extremities (6, 12.8%), the lower extremities (17, 36.2%) and the pelvis (9, 19.1%). Outcomes regarding clinical efficacy, including pain relief, quality of life, and intervention-related complications, were evaluated before and after MWA using the visual analog scale (VAS) and the 36-item Short-Form Health Survey (SF-36) scoring system.

**Results:**

Of the 47 patients included in this study, all of them completed follow-up examinations, with a mean follow-up duration of 4.8 ± 1.6 months (range, 2–9 months). Significantly improved VAS and SF-36 scores were recorded after the initial treatment (P<0.001), suggesting that almost 100% of patients experienced pain relief and an improved quality of life following surgery. No major intervention-related complications (e.g., serious neurovascular injury or infection) occurred during or after the treatment. We recorded only three minor posttreatment complications (6.4%, 3/47), which were related to thermal injury that caused myofasciitis and affected wound healing.

**Conclusion:**

In our study, the short-term efficacy of MWA was considerably favorable, with a relatively low rate of complications. Our results also showed that MWA was effective for pain relief and improved patients’ quality of life, making it a feasible treatment alternative for bone tumors.

## Introduction

Surgical resection and curettage is still considered the mainstay treatment for both benign and malignant bone tumors; however, this type of surgical procedure commonly causes serious physical and emotional trauma for patients [1]. In recent years, minimally invasive modalities have attracted great interest in the field of treating bone tumors; these modalities are associated with low complication rates that may accelerate the recovery of physical function, and patients may accept minimally invasive procedures more readily than open surgery [2]. In general, minor bony lesions, such as osteoid osteoma, enchondroma, and osteoblastoma, are difficult to access surgically and therefore may need large fields of exposure that are out of proportion to the severity of disease and would eventually affect the patients’ ambulation and quality of life [3]. For patients with benign bone tumors, there has been rapid progress in minimally invasive techniques, which are safe, have few complications and offer optimal results [2, 4]. In addition, with progress in cancer therapy and patients living longer with metastatic disease, minimally invasive techniques can also improve pain and quality of life in patients with bone metastases [5]. Several clinical studies have revealed the superior efficacy and safety of minimally invasive surgery for the treatment of benign bone tumors [4, 6] and painful bone metastases [7–9] compared with traditional procedures. Due to the innovations of interventional radiology treatments in clinical practice, various methods for surgical intervention, including radiofrequency ablation (RFA), microwave ablation (MWA), iodine-125 seed brachytherapy, endoscopic techniques, percutaneous vertebroplasty (PVP) and percutaneous kyphoplasty (PKP), have considerably advanced the treatment of bone tumors [6, 10].

Of these methods, MWA is a relatively recent thermal ablation technique with certain theoretical and practical advantages [6, 11]. Compared with other heat-based therapies, this method presents a shorter ablation time, a higher ablation temperature, and a larger ablation zone [11]. In particular, clinical studies have shown that MWA results in lower sensitivity to variations in tissue composition, tissue carbonization and bone impedance, with the advantage that MWA may penetrate tissue more than RFA, to better treat bone tumors with high impedance, such as osseous metastases [12–15].

At present, the treatment of several benign bone tumors, such as osteoid osteomas, with MWA has been shown to be effective [13, 16–18]. However, MWA is a new innovation, and the literature demonstrating its clinical efficiency and safety in treating other benign and malignant bone tumors is relatively sparse. Moreover, the MWA technique has not been widely used for the routine treatment of bone tumors or tumor-like lesions in China. One common reason is that for most nonbone oncology specialized hospitals in China, therapeutic facilities with MWA systems are scarce. Additionally, a truly multidisciplinary approach involving orthopedic oncologists, radiation oncologists, and interventional radiologists is usually ignored, but is essential for diagnostic guidance and treatment evaluation. Clinically, to better verify the feasibility and safety of the MWA technique for the treatment of bone tumors, this study was designed to investigate the short-term clinical performance and safety of MWA for the treatment of both benign and malignant bone tumors and to evaluate the significance of this method in the field of bone tumors.

## Materials and methods

### Ethical approval and consent to participate

All participants and/or their parents were informed of the technique itself as well as possible benefits and complications, and they signed a written consent form for the procedure. All procedures performed in studies involving human participants were in accordance with the ethical standards of the institutional and/or national research committee and with the principles of the 1964 Declaration of Helsinki and its later amendments or comparable ethical standards. The present study was retrospective; for this type of study, the local ethics committee waived formal consent.

### Characteristics of patients

In this study, we retrospectively reviewed the cases of 47 patients who were treated at our department between June 2015 and June 2018. There were 22 males and 25 females, with a mean age of 43.1 ± 16.6 years (range, 8–71 years). The diagnosis of bone tumor was established based on clinical data and imaging studies and confirmed by computed tomography (CT)-guided percutaneous core biopsy or open biopsy before surgery. There were 26 (55.3%) patients with benign bone tumors. Of them, six patients had osteoid osteoma, 5 had osteoblastoma, 12 had enchondroma, 2 had osteofibrous dysplasia, and 1 had a nonossifying fibroma. The remaining 21 (44.7%) had bone metastases (16 patients) or multiple myeloma (5 patients) (Table 1). The primary origin of bone metastases consisted of lung (7 patients), breast (2 patients), liver (2 patients), colorectal (2 patients), cervical (2 patients), and stomach (1 patient) cancer. In addition, the most common lesion location included the lower extremities (*n* = 17, 36.2%), followed by the spine or sacrum (*n* = 15, 31.9%), the pelvis (*n* = 9, 19.1%), and the upper extremities (*n* = 6, 12.8%).

**Table 1:** Patients characteristics (*n* = 47)

Characteristics			No. of patients	Percentage (%)
Age (years)	Mean±SD (years)		43.1±16.6	
	Range		8–71	
Gender	Male		22	46.8%
	Female		25	53.2%
VAS score on admission	<6		20	42.6%
	≥6		27	57.4%
Tumor pathology	Benign bone tumors (*n* = 26)	Osteoid osteoma	6	23.1%
		Osteoblastoma	5	19.2%
		Enchondroma	12	46.2%
		Osteofibrous dysplasia	2	7.7%
		Nonossifying fibroma	1	3.8%
	Malignant bone tumors (*n* = 21)	Bone metastases	16	76.2%
		Multiple myeloma	5	23.8%
Lesion location	Spine or sacrum		15	31.9%
	Upper extremity		6	12.8%
	Lower extremity		17	36.2%
	Pelvis		9	19.1%
Surgical management	MWA		14	29.8%
	MWA+BC		18	38.3%
	Endoscopic MWA+IAB/BC		15	31.9%
Follow-up (months)	Mean±SD (months)		4.8±1.6	
	Range		2–9	

*VAS* Visual analog scale, *MWA* Microwave ablation, *BC* Bone cement, *IAB* Injectable artificial bone

Before undergoing MWA, we usually conduct a multidisciplinary meeting with our experienced oncologists, radiologists and surgeons to assess the preoperative general condition and surgical program of the patients. A significant indication for MWA was clinically significant pain, which was evaluated on a 0–10 visual analog scale (VAS) over the prior 24 h. More than half of these patients (27, 57.4%) had a pain score of 6 or more, which was considered clinically significant. In addition, all patients showed normal or only slightly abnormal levels for heart, liver, kidney and blood functional markers and showed no signs of infection. The life expectancy of patients with bone metastases was more than 6 months according to our assessment. The patients’ health-related quality of life was measured according to the 36-item Short-Form Health Survey (SF-36) scoring system.

### Surgical procedures

#### MWA alone or combined with BC (Fig. 1a-b)

- **Step 1:** Initially, all procedures were performed under local, general or epidural anesthesia guided by CT (SOMATOM Emotion 16; Siemens Healthcare, Erlangen, Germany) or X-ray fluoroscopy. The spine, sacrum and pelvis procedures were usually performed with the patient in the prone position; the extremity procedures were performed with the patient in the supine position. The access path was chosen for the region of interest (ROI), giving priority to the shortest skin-to-target and safest route and avoiding the major blood vessels, the spinal nerve trunk and vital organ structures.
- **Step 2:** One orthopedic surgeon with at least 3 years of experience in interventional bone oncology performed the MWA procedures using a standardized approach. The location of the tumor and the proximity of the tumor to neurovascular nontarget structures are decisive for treatment planning. Once the target lesion and needle path were chosen, the bone was perforated under CT or fluoroscopic guidance using a Paragon bone biopsy system (Paragon Bone Biopsy Systems, Sterylab, Italy), which consists of a 9-gauge external cannula with an internal drill and a 12.5-gauge trephine biopsy needle (Fig. 2d-f, Fig. 3g). Once this procedure was completed, core biopsies were obtained and sent to the laboratory.

**Fig. 1:**
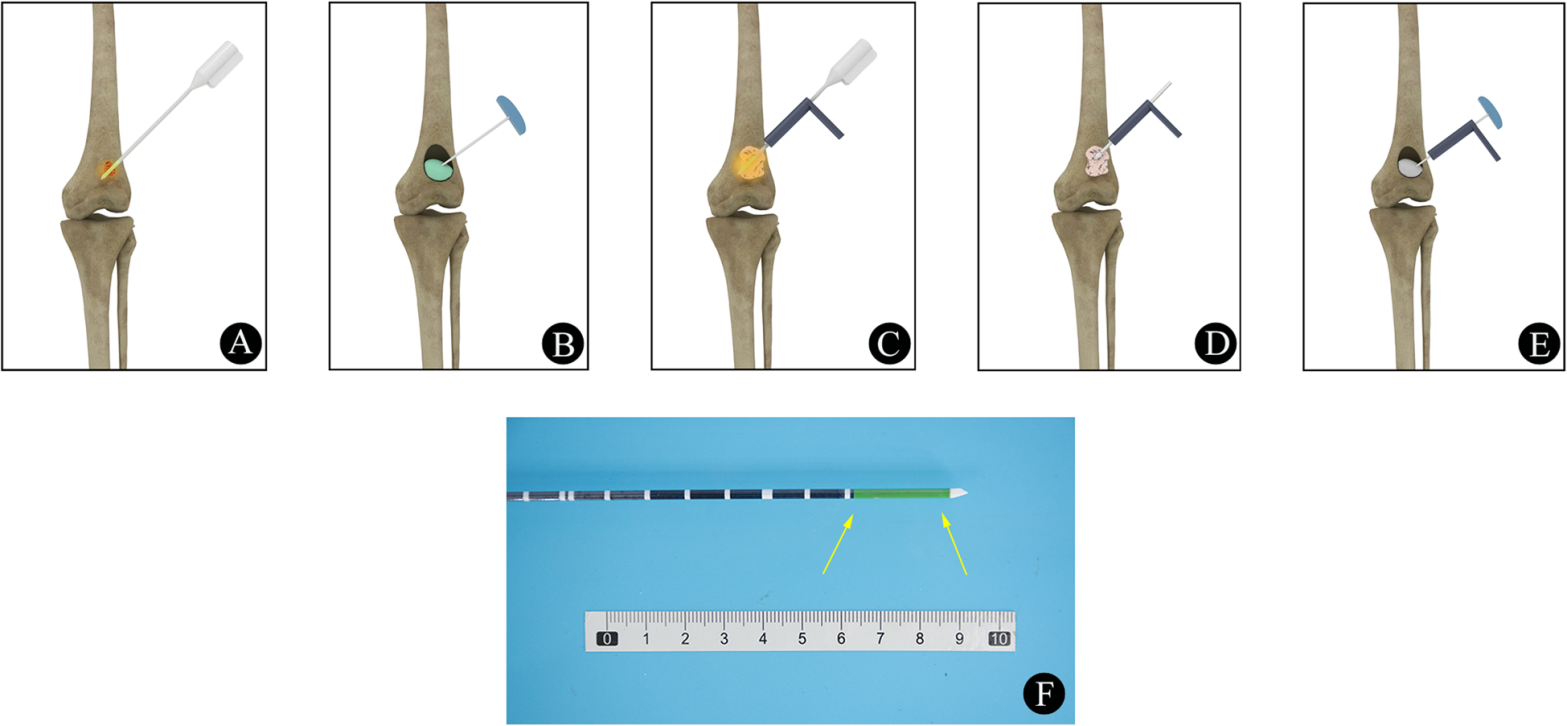
**a**-**b** Schematic diagram of MWA alone (**a**) or combined with BC (**b**). **c**-**e** Endoscopic MWA (**c**) combined with IAB (**e**). **d** Tumor curettage and biopsy. **f** A schematic diagram of the MWA antenna with a 2.8 cm radiating section (yellow arrow)

**Fig. 2:**
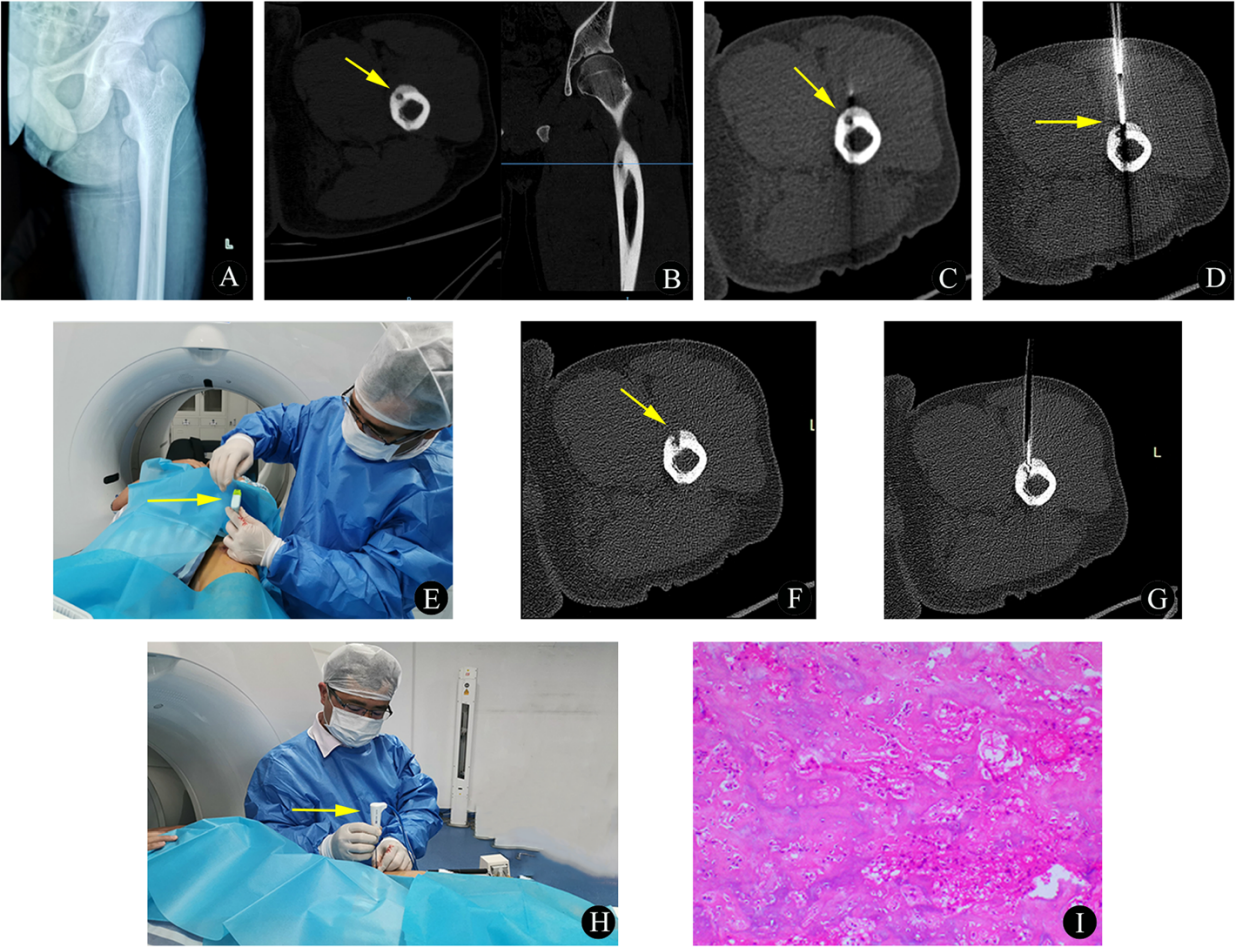
A 25-year-old man with osteoid osteoma of the left femur. **a** Preoperative anteroposterior plain radiographs do not indicate significant neoplastic lesions. **b** The preoperative axial-coronal CT image demonstrates a nidus of the proximal femoral (yellow arrow) with adjacent cortical thickening. **c**-**e** The intraprocedural bone biopsy under CT guidance. The intraprocedural axial CT image (using bone windows) shows that the biopsy needle is inserted into the bone lesion (yellow arrow). **f**-**h** The intraprocedural axial CT image shows the MWA antenna in the center of the lesion. **i** The histopathological biopsy results (hematoxylin and eosin, original magnification 100×) show scattered bone matrix osteoblasts in the tissue; calcification and fibrovascular proliferation were also observed

**Fig. 3:**
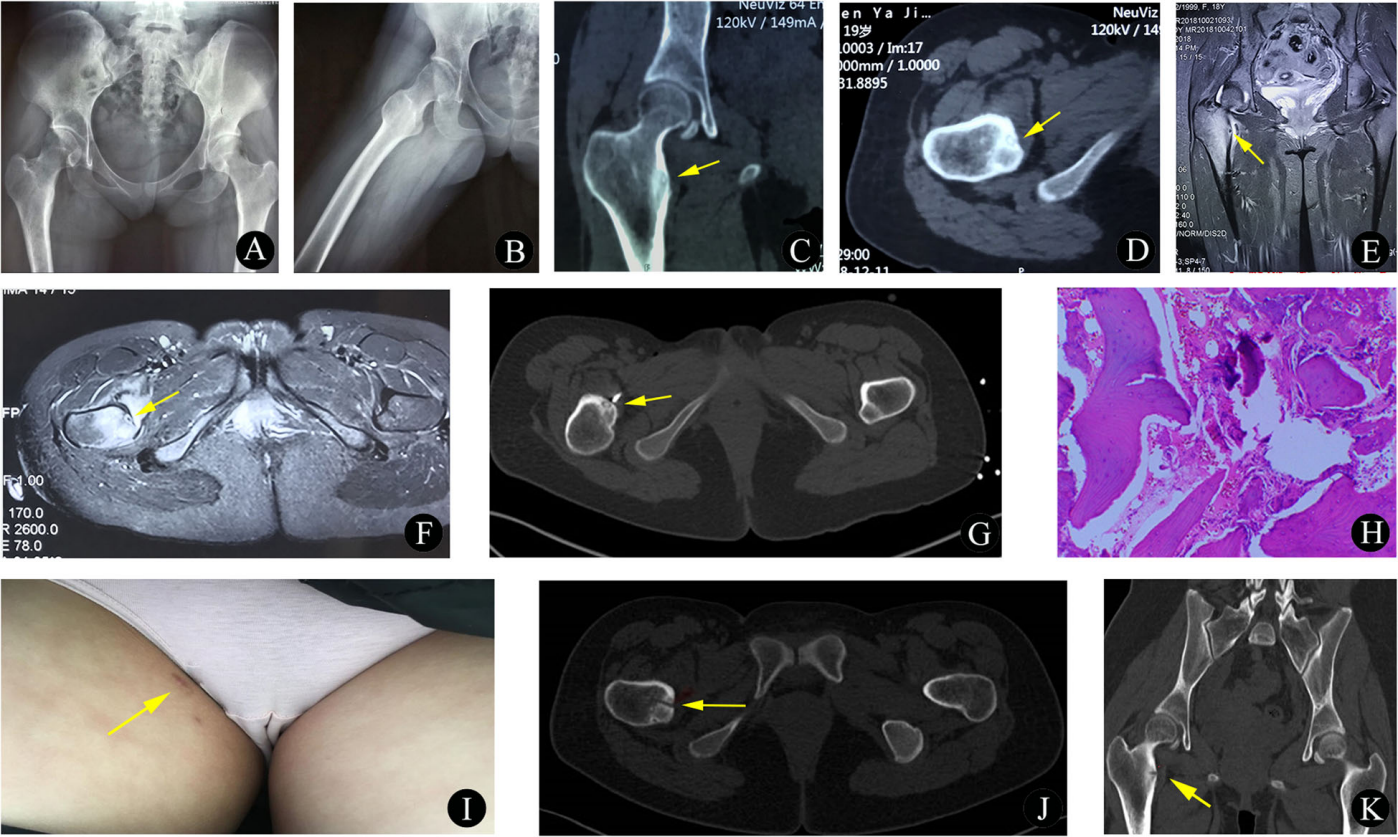
A 19-year-old girl with osteoid osteoma of the right femur. **a**-**b** Preoperative anteroposterior (**a**) and lateral (**b**) plain radiographs do not indicate significant neoplastic lesions. **c**-**d** Preoperative coronal (**c**) and axial (**d**) CT images demonstrate a nidus of the lesser femoral trochanter (yellow arrow) with adjacent cortical thickening. **e**-**f** Preoperative coronal (**e**) and axial (**f**) STIR sequence images show a hypointense central lesion; additionally, slight edema is present in the surrounding soft tissues. **g** The intraprocedural axial CT image shows that the MWA antenna was inserted into the bone lesion (yellow arrow) and that the tumor tissue was reached. **h** The histopathological biopsy results (hematoxylin and eosin, original magnification 100×) diagnosed the bone lesion as an osteoid osteoma. **i**-**k** At the 3-month follow-up visit, wound recovery (**i**) was satisfactory, the tumor was successfully removed after ablation, and no significant progression of the lesion was observed on the CT scan (**j**-**k**)

**Fig. 4:**
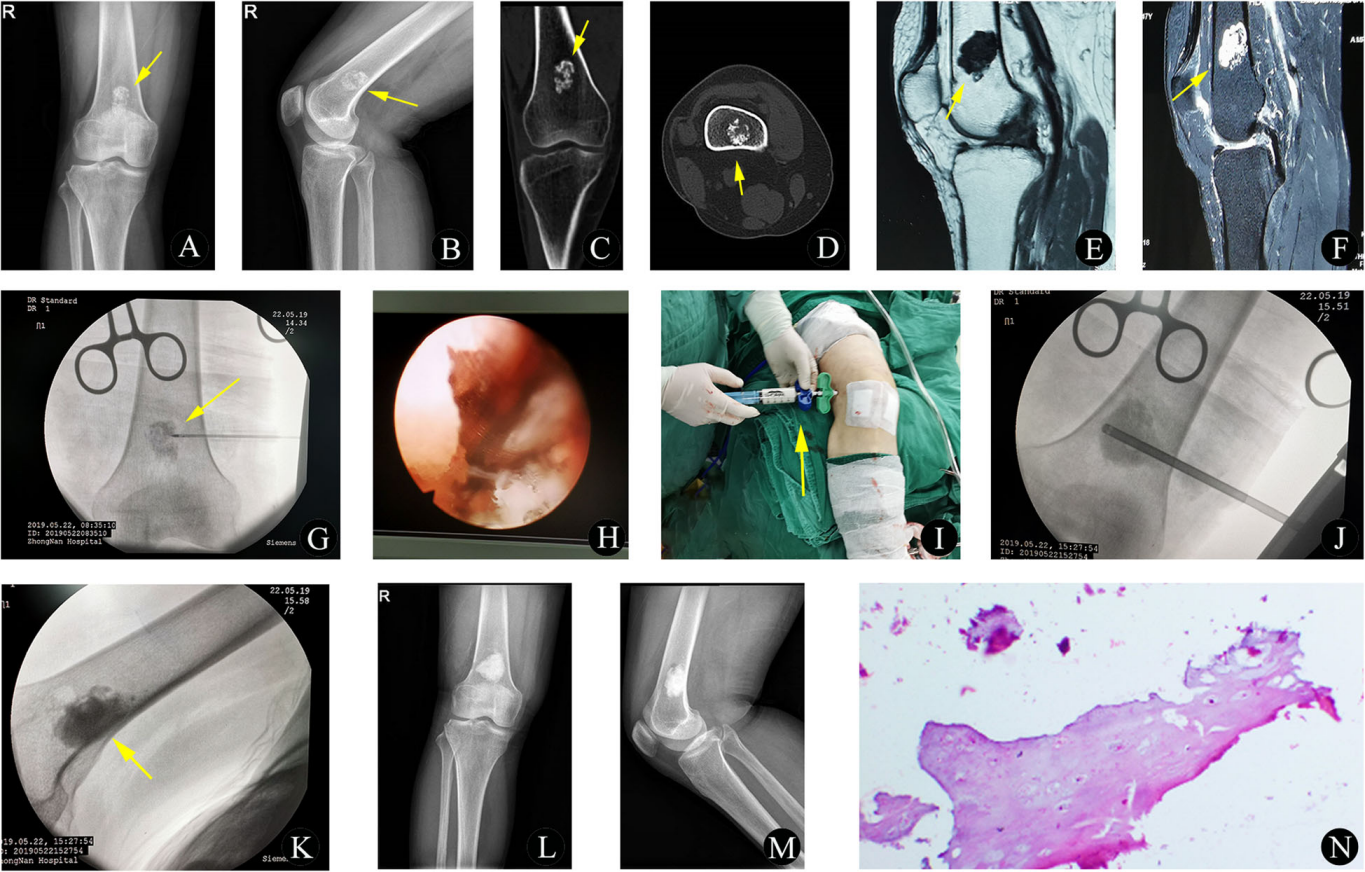
A 46-year-old woman with enchondroma of the right femur. **a**-**b** Preoperative anteroposterior (**a**) and lateral (**b**) plain radiographs show a central bone lesion with local high-density shadows in the distal femur (yellow arrow). **c**-**d** Preoperative coronal (**c**) and axial (**d**) CT images demonstrate the mass as a high-density lesion without a definite boundary in the distal femur (yellow arrow). No obvious periosteal reaction or soft tissue masses were found in the local cortex. **e**-**f** Preoperative sagittal T1-weighted (**e**) and sagittal STIR sequence (**f**) images show a central lesion with low signal intensity on T1WI and heterogeneous high signal intensity on STIR (yellow arrow). **g**-**h** Intraoperative X-rays showing the MWA procedure (yellow arrow). Subsequently, endoscopic curettage of the tumor was performed. **i**-**k** The remaining bony cavity was packed with injectable artificial (**i**, yellow arrow) bone under the guidance of fluoroscopy (**k**, yellow arrow). **l**-**m** At the 5-month follow-up visit, solid fusion of the bone graft at the distal femur without tumor recurrence was observed. **n** The histopathological biopsy results (hematoxylin and eosin, original magnification 40×) confirmed the bone lesion as an enchondroma

Subsequently, a single 17-gauge, liquid-cooled antenna with a 2.8-cm radiating section was coaxially inserted into the tumor (Fig. 1f, Fig. 2g-h). The microwave generator used was a 2450 ± 50 MHz generator with an output power ranging from 5 to 100 W. (Emprint™ Ablation System with Thermosphere™ Technology, Covidien, Shanghai, China). For most benign bone tumors, a 3–6 min application at 60 W was standard, according to our expertise in MWA for the treatment of bone tumors (Figs. 2, 3). For malignant bone tumors, a minimum ablation cycle of 5 min was performed with a target temperature of 60 °C. Each ablation cycle lasted 30 s. However, depending on the size and location of the lesion, we adapted the power settings and ablation time based on the manufacturer’s recommendations. Immediately after the MWA procedure, bone cement (BC) was applied according to the tumor size and appropriate injection site (Fig. 4g-j).

- **Step 3:** After the removal of the antenna, manual compression was applied for 3–5 min at the puncture site. All patients were observed for at least 3 h after the procedure to ensure hemodynamic stability, and their respiratory condition was monitored. Then, all patients were allowed to mobilize gradually, and in the case of lower extremity lesions, full weight-bearing was contraindicated for a 1-month period.

#### Endoscopic MWA combined with IAB or BC (Fig. 1c-e)

**Fig. 5:**
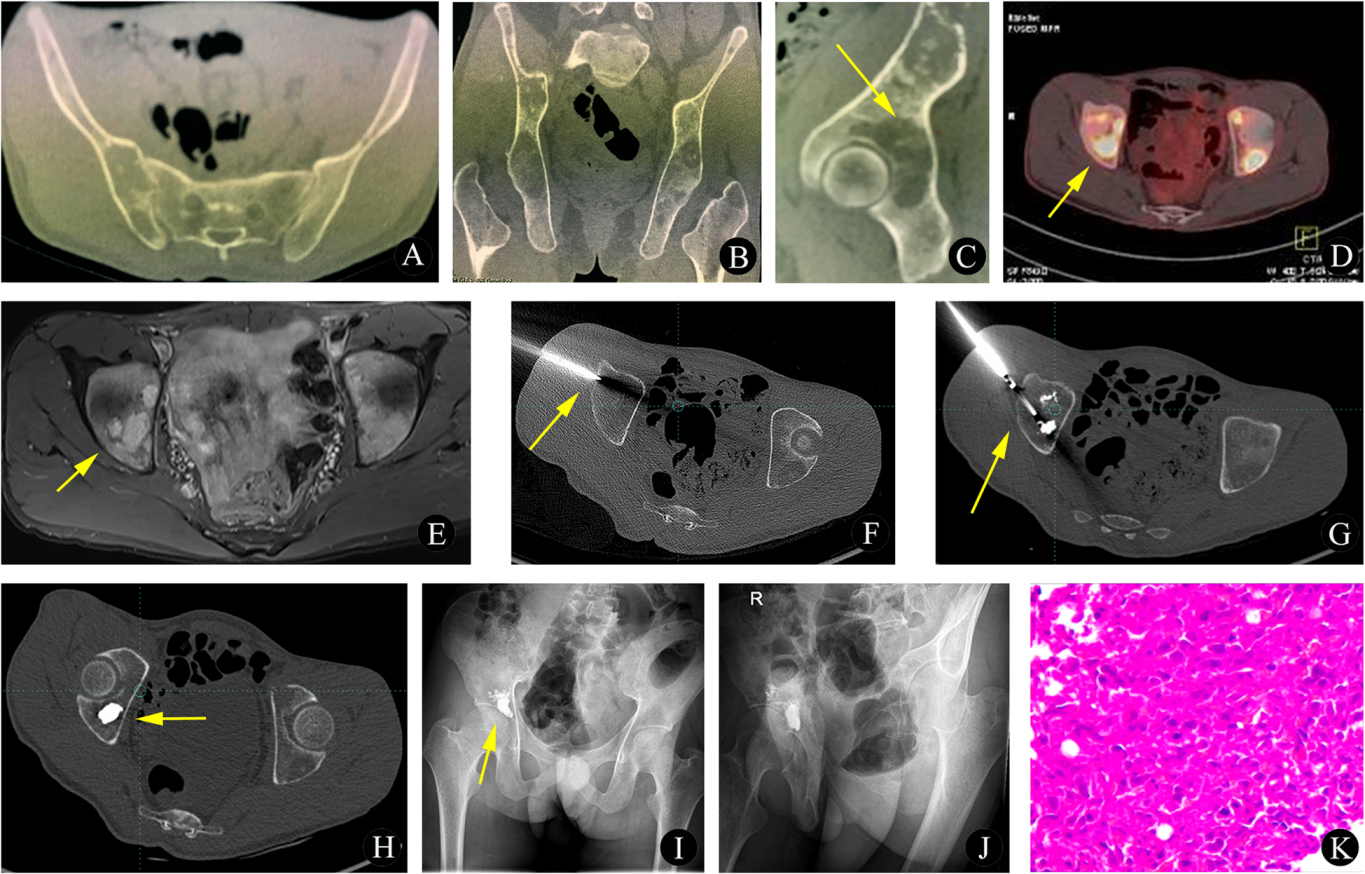
A 27-year-old man with bone metastasis of gastric cancer. **a**-**c** Preoperative CT images show multiple sites of bone destruction in the pelvis, especially the acetabulum (yellow arrow). **d** Preoperative ^18^F-FDG PET/CT imaging shows uptake in the bone lesion in the acetabulum (yellow arrow). **e** The preoperative axial STIR sequence shows multiple heterogeneous high signal intensities with soft tissue masses (yellow arrow). **f**-**h** Intraoperative MWA and BC filling (yellow arrow). **i**-**j** At the 3-month follow-up visit, there was solid fusion of the BC without infiltration of the articular cavity (yellow arrow). **k** The histopathological biopsy results (hematoxylin and eosin, original magnification 100×) confirmed that the lesion was a bone metastasis from gastric cancer

**Fig. 6:**
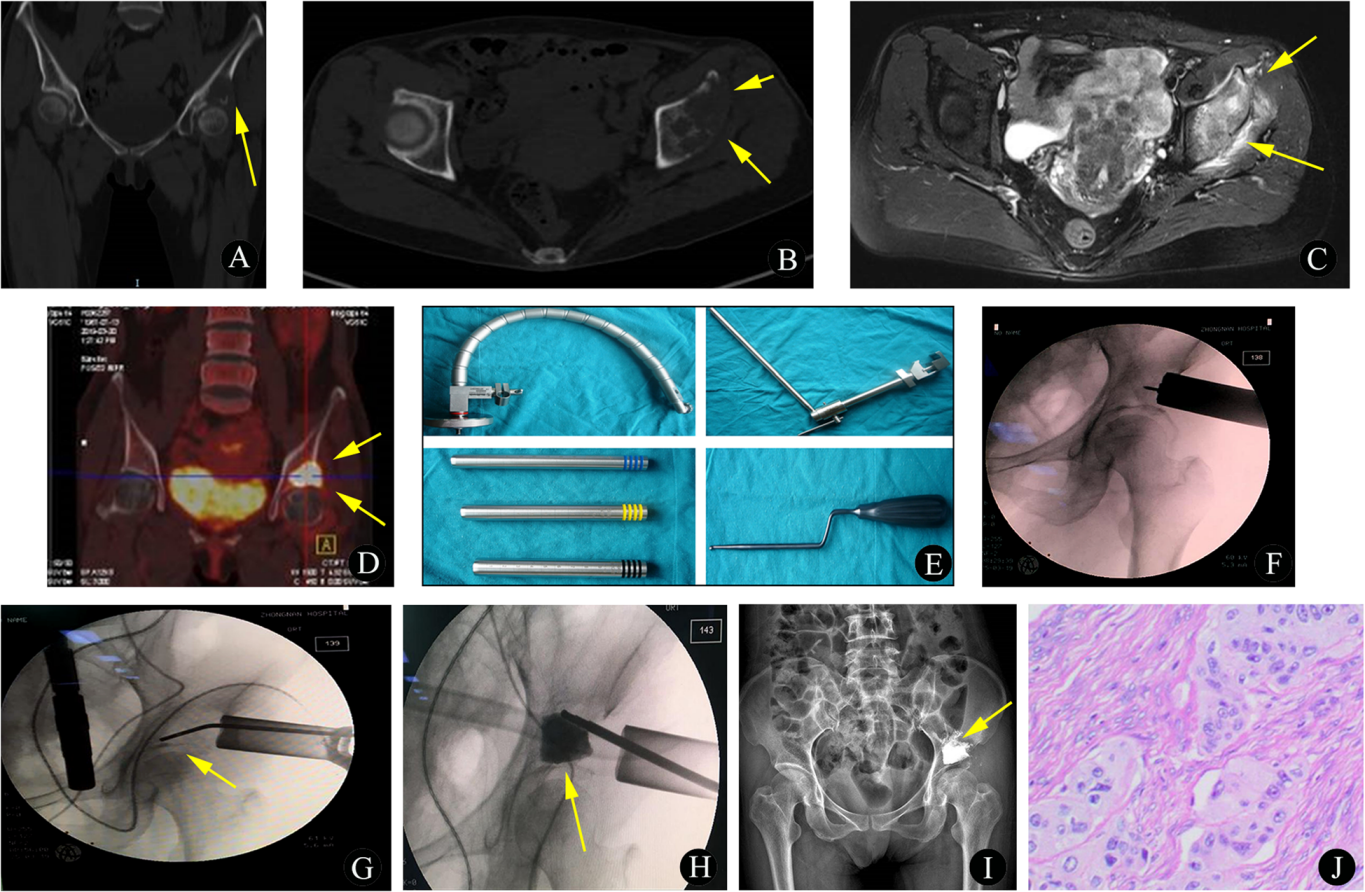
A 51-year-old woman with bone metastasis from lung cancer. **a**-**b** Preoperative coronal (**a**) and axial (**b**) CT images demonstrate osteolytic destruction of the left acetabulum (yellow arrow). **c** The preoperative axial STIR sequence shows a heterogeneous high-intensity signal with a soft tissue mass (yellow arrow). **d** Preoperative ^18^F-FDG PET/CT imaging shows uptake in the bone lesion in the left acetabulum (yellow arrow). **e** A schematic diagram of the Mast-Quadrant minimally invasive system. **f**-**g** Intraoperative MWA through endoscopic channels (yellow arrow). **h**-**i** Intraoperative BC filling. **j** The histopathological biopsy results (hematoxylin and eosin, original magnification 40×) confirmed that the lesion was a bone metastasis from lung cancer

After the determination of the target lesion and needle path, a MAST QUADRANT minimally invasive system (Fig. 5e-h) was utilized. In detail, a 3-cm incision was made on the surface over the lesion. Then, the dilating catheter was inserted step by step through a hook wire, and the soft tissue was gradually separated. The dilating catheter was preferably placed at the thinnest points of the affected cortical bone. When the surgical approach was well established, the dilating catheter was removed, and a 5-mm drill was used for cortical bone fenestration. All of these procedures were monitored using an intervertebral foraminoscope (Fig. 6h). The tumor tissue could then be biopsied with biopsy forceps, subjected to MWA with antenna, and curetted with an arthroscopic curette (Fig. 1d). After the above procedures were completed, the remaining bony cavity could be packed with injectable artificial bone (IAB) material (Osteolink Biomaterial Co., Ltd. Hubei, China) (Fig. 1e, Fig. 6i-m) or BC as needed, according to the type of tumor, and the application of IAB material or BC was guided by radiography (Fig. 5f-h). All of these procedures were performed by the same team with least 3 years of ablation experience in interventional bone oncology.

### Follow-up and clinical evaluations

The follow-up radiological assessment was performed before and 1 month after ablation and then every 3 months for the first 2 years. Medical imaging, including standard radiography and CT or magnetic resonance imaging (MRI), was performed in all of the patients. CT- or fluoroscopy-guided percutaneous biopsy was performed before ablation and used for a pathological diagnosis. In addition, clinical data were reviewed in all cases to monitor pain relief, improvement in quality of life, and procedure-related complications. Pain management was assessed via an ordinal VAS scoring system ranging from 0 to 10, where 0 represents no pain, and 10 represents the worst possible level of pain. The SF-36 survey was used to measure health-related quality of life before and after treatment. The clinical outcomes were assessed preoperatively and at the last follow-up visit using the VAS score, incidence of complications, and the SF-36 score. Any complications related to the treatment were recorded.

### Statistical analysis

All continuous variables are expressed as the mean ± SD. Qualitative variables are expressed as numbers and percentages. A paired t-test was adopted to compare the preoperative and final follow-up VAS and SF-36 scores. The statistical significance was set at *P* < 0.05. All statistical analyses were completed using SPSS (version 22.0; IBM, Chicago, IL, USA).

## Results

**Fig. 7:**
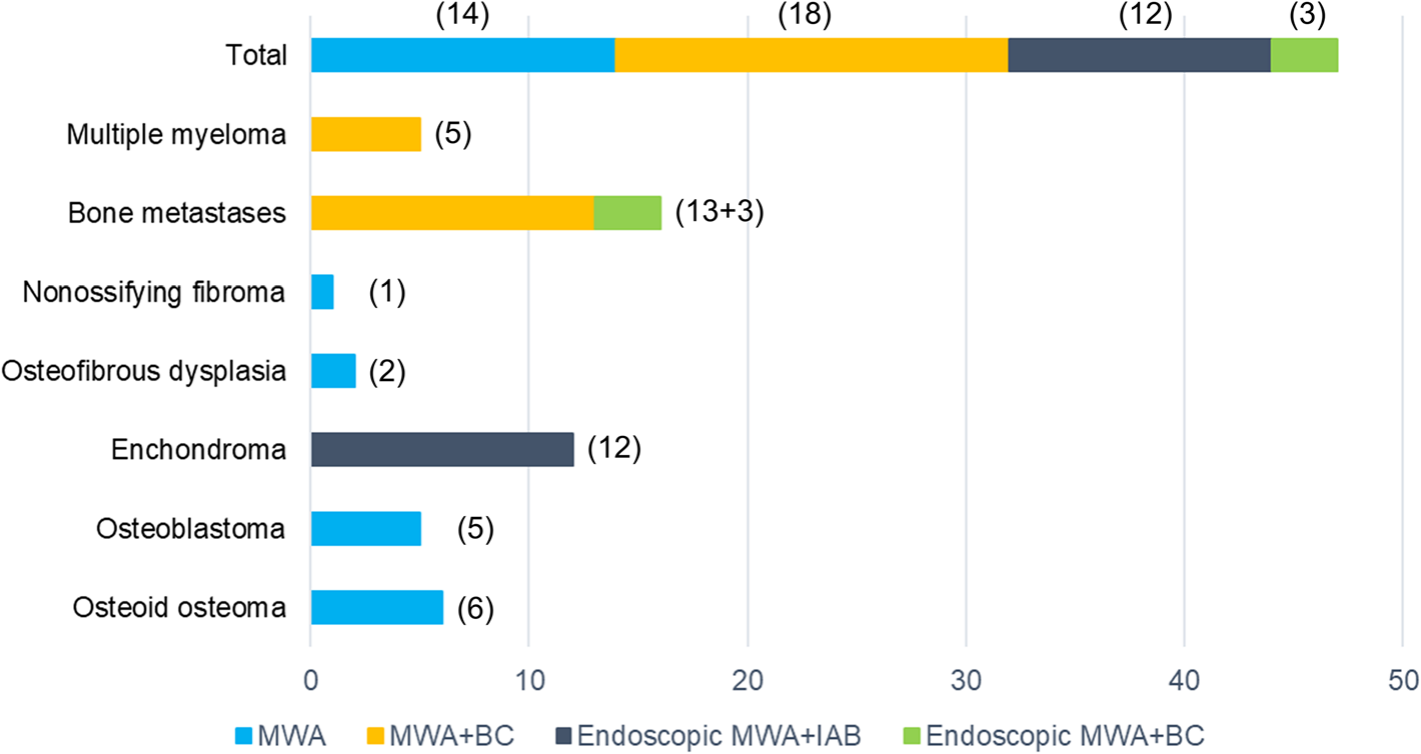
Surgical details of the application of MWA for the treatment of bone tumors

**Fig. 8:**
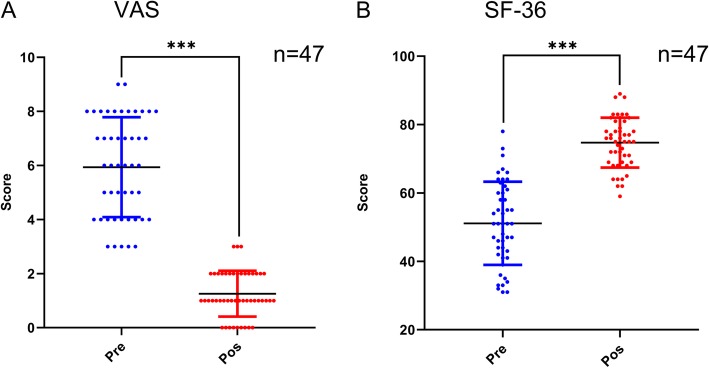
**a**-**b** VAS and SF-36 scores for all patients preoperatively and at the final follow-up visit. VAS, visual analog scale; SF-36, the 36-item Short-Form Health Survey; Pre, preoperative; Pos, postoperative (at the final follow-up) follow-up visit. ***P < 0.001

In this study, all patients completed percutaneous MWA and the postoperative clinical follow-up examinations. A total of 47 patients (100%) were identified, with an average follow-up duration of 4.8 ± 1.6 months (range, 2–9 months). No patients were lost to follow-up. Of these, the majority of patients (18, 38.3%) were treated with MWA and BC, followed by endoscopic MWA and IAB or BC (15, 31.9%), and MWA alone (14, 29.8%). The clinical data and surgical details are shown in Table 1 and Fig. 7. At the final follow-up, the VAS scores were less than the values observed preoperatively for all patients, and the differences between the 2 time points were statistically significant (*P* < 0.001). Meanwhile, excellent and improved quality of life was observed according to the SF-36 scoring system for all patients (Fig. 8), and the difference between the preoperative and the final follow-up scores was statistically significant (*P* < 0.001).

In addition, we evaluated the clinical outcomes between patients with benign tumors and those with malignant bone tumors. As shown in Table 2, for patients with benign bone tumors, a significant decrease in pain perception was documented during the follow-up period, and the VAS score decreased from 4.9 ± 1.7 to 0.8 ± 0.7 (*P* < 0.001). The SF-36 score also improved from 58.1 ± 9.4 before surgery to 78.7 ± 5.2 at the last visit, reflecting significant recovery (*P* < 0.001). Moreover, the same results were observed for patients with malignant bone tumors.

**Table 2:** Preoperative and postoperative data regarding surgical efficacy according to the VAS and SF-36 scores

	VAS score				SF-36 score			
	Pre	Pos	t value	*P* value	Pre	Pos	t value	*P* value
BBT (*n* = 26)	4.9 ± 1.7	0.8 ± 0.7	11.984	<0.001	58.1 ± 9.4	78.7 ± 5.2	−15.37	<0.001
MBT (*n* = 21)	7.2 ± 1.1	1.8 ± 0.7	22.076	<0.001	42.5 ± 9.4	69.7 ± 6.4	−18.62	<0.001

*Pre* Preoperatively, *Pos* Postoperatively, *VAS* Visual analog scale, *SF-36* 36-item Short-Form Health Survey, *BBT* Benign bone tumor, *MBT* Malignant bone tumor

No major intervention-related complications (e.g., serious neurovascular injury, fracture or infection) occurred during or after the treatment. However, minor complications, which were related to thermal injury that caused myofasciitis and affected wound healing, occurred in 3 patients (6.4%). During the follow-up period, no other complications or deaths occurred among our study population.

## Discussion

### Advantages of MWA for the treatment of bone tumors

The main finding of our study is that MWA can effectively relieve pain and improve the quality of life of patients with bone tumors, including benign or malignant bone tumors, and a significant difference was observed between preoperative and final follow-up measures. Reducing pain and improving quality of life are the primary goals in the treatment of bone tumors by multidisciplinary care teams [19]. With particular reference to ablation, MWA is a local thermal ablation technique that uses a rapidly oscillating electromagnetic field to cause water molecules to rotate, resulting in frictional heating [6]. Depending on the procedure, the surgeon can place an ablation antenna in the tumor to induce coagulation necrosis in the target tissue under imaging guidance. In recent years, MWA has been shown to be a safe and clinically efficacious treatment for liver cancer, lung cancer and other clinical fields [11]. However, clinical evidence associated with the application of MWA for the treatment of bone tumors has been limited to some small series [11, 13, 16–18, 20–22]. In a prospective study by Prud’homme et al. [13], thirteen cases of osteoid osteoma of the extremities were treated by MWA under CT guidance. During the follow-up period, the overall success rate was up to 92.3% (12/13), and almost all patients experienced total pain relief. Recently, Deib et al. [21] performed the largest retrospective study of painful extraspinal osseous metastases and myelomatous tumors treated with MWA and BC. They concluded that MWA is a promising, safe, and effective treatment for painful spinal metastasis that can result in both a reduction in pain and a degree of local control over the disease process. Moreover, in a retrospective study, Khan et al. [22] reported on the use of MWA to treat 102 painful spinal metastases (69 patients), which included spinal lesions in 12 patients. Local tumor control was achieved in all patients, and significant pain palliation was achieved at 2–4 weeks and 20–24 weeks following the procedure. Our results are in line with those of the above mentioned previous study and further confirm the excellent curative potential of MWA.

### Application of MWA in benign bone tumors

Clinically, percutaneous RFA is a well-established gold standard for treating osteoid osteoma and other benign bone lesions [6, 8, 9, 12, 14, 19]. In RFA, a needle electrode is inserted into a target zone, and heat generated by dielectric heating at the needle tip causes coagulation necrosis of the bone lesion. However, poor thermal conduction through bone is a limiting factor in RFA. In tissues with a higher impedance, such as bone, there is a reduction in energy deposition from RFA, which, in turn, leads to a smaller temperature increase and a potential increase in the treatment failure rate [12, 16, 21]. Interestingly, the characteristics of MWA can overcome the limitations of RFA, leading to a considerably improved power efficiency and the rapid coagulative necrosis of tumor cells. In addition, MWA can produce faster heating, higher intralesional temperatures, and less susceptibility to both heat-sink and charring effects. It is also fairly insensitive to the intrinsic high impedance of bone, especially in the case of osteosclerotic tumors, as it allows deeper thermal penetration than other modalities [6, 21, 22]. According to animal models, Brace et al. [23] established that the application of MWA is more advantageous in bone tissue with high impedance. Based on these advantages, we successfully applied MWA in the treatment of benign bone lesions, including osteoid osteoma, osteoblastoma, enchondroma, osteofibrous dysplasia and nonossifying fibroma. In detail, MWA alone was performed in 14 patients (6 with osteoid osteoma, 5 with osteoblastoma, 2 with osteofibrous dysplasia, and 1 with nonossifying fibroma), and the remaining 21 patients with enchondroma underwent endoscopic MWA combined with IAB. It is noteworthy that enchondroma can most often be adequately treated with intralesional curettage and bone grafting [24]. To reduce the surgical trauma and achieve better local control over the tumor, we designed our endoscopic MWA technique combined with IAB to sustain tumor necrosis and restore structural stabilization. In 2015, Lui et al. [25] reported a technique consisting of endoscopic curettage and bone grafting for treating enchondroma of the proximal phalanx of the hallux; complete incorporation of the bone graft and a good range of motion of the hallux were achieved 3 months after the operation. In our study, with the help of a MAST QUADRANT minimally invasive system, we carried out the MWA and curettage of tumor tissue under direct vision, completely removed the tumor tissue, and repaired the bone defects at the same time. Our results are consistent with the results of preliminary studies evaluating MWA in the treatment of benign bone tumors [13, 16, 17].

### Application of MWA in malignant bone tumors

In general, bone metastases commonly occur in patients with advanced disease. The treatment of bone metastases is typically aimed at both pain relief and the preservation of ambulatory functions. Percutaneous MWA is an effective alternative modality for relieving the pain of patients with bone metastases, particularly when patients with metastatic disease are often undertreated for pain [21, 22]. However, MWA alone may render cavity formation and bone mass reduction, resulting in an increased risk of pathological fractures. Meanwhile, some scholars believe that thermal ablation can create a cavity through tissue dissolution rather than tissue displacement alone, leading to cement deposition and hence theoretically minimizing the risk of complications [26]. Therefore, MWA combined with BC may be a useful method for both additional pain relief and structural stabilization in the treatment of bone tumors [27]. Both Deib et al. [21] and Khan et al. [22] have described how MWA can be performed through safe, repeated, short ablation cycles to control the diffusion of the heating zone without diminishing the efficacy of MWA. In the current study, all patients with malignant bone tumors, including bone metastases and multiple myeloma, were treated with the application of BC. Of these, 13 patients with bone metastases and 5 with multiple myeloma were treated with MWA combined with BC, and the remaining 3 patients with bone metastases were treated with endoscopic MWA combined with BC. According to our experience, bone tumors located in the pelvis may present different challenges to the treating physician due to complex anatomical structures. Furthermore, pelvic metastatic tumors may be particularly painful and debilitating [21]. It seems possible that endoscopic MWA combined with BC can be performed through safe and short ablation cycles to control the diffusion of the heating zone without damaging vital nerves or blood vessels. Recently, Fan et al. [28] attempted en bloc MWA in situ to improve the outcome of the treatment of primary malignant pelvic bone tumors, with encouraging oncological and functional results. Our results are consistent with those of previous studies, with immediate pain reduction and quality of life improvement obtained in almost 100% of the patients and maintained at the final follow-up visit.

Nonetheless, how to evaluate changes in bone and soft tissue after RFA or MWA remains one of the most challenging issues for oncologic orthopedic surgeons and radiologists. Recently, Razek and his colleagues reported a series of cases with benign and malignant bone tumors that underwent diffusion-weighted MR imaging (DWI). Those investigators concluded that different tumor tissues have different DWI findings and different apparent diffusion coefficient (ADC) values. Inspired by the discovery of these different ADC values, we believe that DWI may provide essential information for assessing the tissue changes following tumor removal by RFA or MWA [29–32].

### Complications

Percutaneous MWA techniques have been used for the treatment of patients with several benign and metastatic bone lesions, and major complications are infrequent [6, 15–17, 21, 22, 27, 33, 34]. Kastler et al. [27] reported the successful treatment of spinal metastases with MWA, without any major complications. Subsequently, in 2017, Kastler et al. [35] also reported the use of a thermocouple technique for real-time temperature control during MWA in the treatment of metastatic bone disease. In their series, the maximum temperature of the thermocouple near the monitored root did not exceed 43 °C, which served as an added safety feature. Furthermore, light sedation or local anesthesia adds to the safety of the procedure because patients may alert the surgeon in the case of pain. Recently, Cheng et al. [34] have shown that ultrasound-guided percutaneous MWA has the advantages of being a real-time, convenient, lower-cost and nonradiative treatment; additionally, complications related to thermal damage (in the form of skin burns), infection and nerve injury did not occur in any patients. Other recent and mostly retrospective studies have shown the same results, with a relatively low rate of complications [11, 15, 16, 21, 22]. Similarly, the results of our study also indicate that MWA is a safe and efficient approach, with only three minor complications related to thermal injury that caused myofasciitis and affected wound healing in the entire study. However, some scholars would have preferred the more diffuse RFA technology over MWA, with poor ablation zone predictability, which may present an increased risk of complications, especially along the antenna. Indeed, early MWA technologies may present greater inherent limitations. In the current study, we utilized an MWA system based on Thermosphere™ Technology, and newer probes have solved this major performance issue, enabling the more precise delivery of energy to the tissue with consequent large, spherical and predictable ablation zones [36].

Limitations to this study must be acknowledged. First, this was a retrospective observation from a single institution in a population of Chinese patients. However, our primary goal was to demonstrate the feasibility and safety of MWA in the treatment of bone tumors, including benign and malignant bone lesions. A higher level of evidence could be achieved by performing a prospective, multicenter trial in the future. Second, this study does not constitute a comparative study, as no other thermal ablation techniques (e.g., RFA, laser ablation, and cryoablation) were observed, and no long-term follow-up data were assessed. In addition, various subtypes and volumes of bone tumors present differences in terms of MWA technique. In regard to these problems, more randomized studies will gradually be conducted in the future.

## Conclusion

In our study, the short-term efficacy of percutaneous MWA is considerably favorable, with no major complications occurring among the selected patients in the current study. Our results also show that MWA is effective for pain relief and improved the quality of life, making it a feasible, safe, and effective treatment alternative for bone tumors. However, further investigations are needed to assess the clinical efficacy of this technique compared with that of other existing techniques.

## Data Availability

The datasets used and/or analyzed during the current study are available from the corresponding author on reasonable request.

## Abbreviations

BC: Bone cement
CT: Computed tomography
FDG: ^18^F-fluorodeoxyglucose
IAB: Injectable artificial bone
MRI: Magnetic resonance imaging
MWA: Microwave ablation
PET/CT: Positron emission tomography/computed tomography
PKP: Percutaneous kyphoplasty
PVP: Percutaneous vertebroplasty
RFA: Radiofrequency ablation
SF-36: 36-item Short-Form Health Survey
VAS: Visual analog scale
